# Transformation with Oligonucleotides Creating Clustered Changes in the Yeast Genome

**DOI:** 10.1371/journal.pone.0042905

**Published:** 2012-08-14

**Authors:** Gina P. Rodriguez, Joseph B. Song, Gray F. Crouse

**Affiliations:** 1 Department of Biology, Emory University, Atlanta, Georgia, United States of America; 2 Winship Cancer Institute, Emory University, Atlanta, Georgia, United States of America; University of Minnesota, United States of America

## Abstract

We have studied single-strand oligonucleotide (oligo) transformation of yeast by using 40-nt long oligos that create multiple base changes to the yeast genome spread throughout the length of the oligos, making it possible to measure the portions of an oligo that are incorporated during transformation. Although the transformation process is greatly inhibited by DNA mismatch repair (MMR), the pattern of incorporation is essentially the same in the presence or absence of MMR, whether the oligo anneals to the leading or lagging strand of DNA replication, or whether phosphorothioate linkages are used at either end. A central core of approximately 15 nt is incorporated with a frequency of >90%; the ends are incorporated with a lower frequency, and loss of the two ends appears to be by different mechanisms. Bases that are 5–10 nt from the 5′ end are generally lost with a frequency of >95%, likely through a process involving flap excision. On the 3′ end, bases 5–10 nt from the 3′ end are lost about 1/3 of the time. These results indicate that oligos can be used to create multiple simultaneous changes to the yeast genome, even in the presence of MMR.

## Introduction

It was first demonstrated in the Sherman lab that single-stranded oligonucleotides (oligos) could be introduced into yeast cells and create permanent changes in the genome, although the mechanism of that transformation was not understood [1]–[3]. Later work in both *E. coli* and yeast, in which events can be determined in relation to known origins of replication, has shown that transformation is more efficient when oligos anneal to the lagging strand [4], [5]. Our work [5], [6] as well as others [7] strongly supports the view that oligo transformation is generally due to the incorporation of the oligo at the replication fork and the oligo then serves as a primer for continued replication.

The early experiments used strains proficient in mismatch repair (MMR). One issue has been the role of MMR in the transformation process. MMR recognizes DNA mismatches created in the process of replication and removes bases on the primer strand, using some sort of strand discrimination process such as nicks on the newly replicated strand to determine which strand to remove [8]–[10]. There are two recognition complexes in most eukaryotes, MutSα, a heterodimer of Msh2 and Msh6 that recognizes base-base mismatches and small loops, and MutSβ, a heterodimer of Msh2 and Msh3 that recognizes loops [8]–[10]. MMR is also important in preventing recombination between non-identical DNA [8]–[10]. Although that process of heteroduplex rejection is not coupled to replication [11], the invading strand is preferentially eliminated. Because the function of MMR is to remove newly replicated DNA or invading DNA that would create mismatches with the existing DNA, one might have expected MMR to interfere with the process of oligo transformation. However, it was postulated that the transformation process in yeast was a gene correction event requiring MMR [12] and later work indicated that Msh2 assisted transformation in yeast, but hindered transformation in mammalian cells [13]. In contrast, experiments in *E. coli* [4], [14] and our experiments in yeast [5] both demonstrate a strong blocking effect of MMR, consistent with its activity both in preventing replication errors and heteroduplex rejection. There seems to be general agreement that oligo transformation in mammalian cells is blocked by MMR [7], [15], [16].

With a few exceptions [17]–[19], experiments with oligo transformation in eukaryotes have examined only the change of contiguous bases, mediated usually by bases in the center of a transforming oligo. Early work from the Sherman lab found that transformation decreased as the number of central mismatches increased, except for a situation in which there were 9 mismatches in a row [2]. That puzzling result can now be understood as the relative invisibility of a large loop to MMR [5], [16]. The Sherman lab also found that mismatches at either end of an oligo were not incorporated [2]. One study in yeast found that sites 4 bases apart were usually incorporated together, whereas a separation of 15 or more bases apart led to low frequencies of simultaneous incorporation [19]. A report of oligo transformation in mammalian cells found that sites separated by 14 bases could be simultaneously incorporated but that the percentage of co-incorporation appeared to decrease linearly with distance [17].

Our interest in this study was to better understand the mechanism of incorporation of oligos into the genome and in particular the pattern of incorporation throughout the length of the oligo. There could well be biases in 5′ versus 3′ end incorporation, and on whether MMR were present and on which strand of replication an oligo were incorporated. One of our future interests is to use oligos to incorporate specific damaged bases into the genome for subsequent analysis, and for such studies it would be particularly important to know what regions of an oligo were likely to be incorporated. In addition, there has been considerable interest in oligo transformation as a means of gene therapy, and understanding the process is crucial to its use [7].

## Materials and Methods

### Yeast Strains

**Figure 1:**
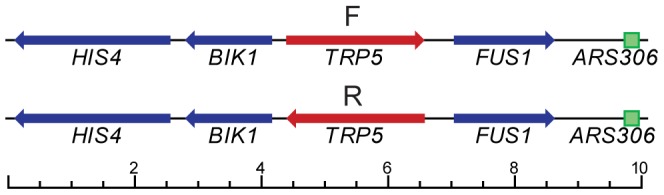
Location of *TRP5* gene in G148Cm F and R strains. The *TRP5* gene, in both orientations, replaced the *RNQ1* gene on Chromosome III near the *ARS306* origin of replication as illustrated above. Shown below is a scale of distance in kb.

The *trp5* G148Cm mutation was created as described for the other *trp5* mutations [20] using *delitto perfetto* [21] and created the sequence C GATGTTATCCAACTGGGA starting at position 138 of *TRP5* with mutated bases underlined. The location of the *TRP5* gene in both orientations with respect to the surrounding genes and the nearby *ARS306* origin of replication is illustrated in Fig. 1. The *lys2CT_1265_GA* mutation was similarly created by *delitto perfetto*. The genotypes of strains used in these experiments is given in Table S1. All gene deletions were created by one-step disruption with PCR generated fragments. In general gene deletions were made from a PCR fragment generated from the collection of yeast gene deletions [22]. The kanMX4 resistance marker was changed to hphMX4 by transformation with a fragment from pAG32 [23].

### Yeast Transformation

Transformation was as described previously [6]. For a typical transformation, 200 pmol of a Trp oligo and 200 pmol of LYS2TCARev40 was used for a 200 µl of this cell suspension. Immediately after electroporation, the cell suspension was added to 5 ml YPAD, and the cells incubated at 30° with shaking for 2 h. Cells were then centrifuged, washed with H_2_O, and plated on synthetic dextrose (SD) medium lacking either tryptophan or lysine [24] to select transformants. The Lys oligo was originally designed to be used as an internal control for transformation. However, it was subsequently found that the number of Lys+ transformants was not correlated well enough with the number of Trp+ transformants to be used as an internal control (results not shown). The sequence of all oligos used is given in Table S2.

### PCR and Revertant Analysis

Individual Trp+ revertants were picked into 200 µl SD-Trp medium in 96-well deep-well plates, grown overnight at 30° with shaking, a small aliquot of each transferred to fresh SD-Trp medium with a Boekel Microplate Replicator and grown overnight again, and finally transferred with the replicator to another deep-well plate for overnight growth in 300 µl YPAD. Cells were then transferred with the replicator to a PCR microplate containing 15 µl per well of 2 mg/mL Zymolyase 20T (USBiological) in 0.1 M Phosphate Buffer pH 7.4 and incubated at 37° for 30 min and 95° for 10 min. After incubation, 85 µl H_2_O was added to each well. PCR was performed using 5 µl of the lysate in a total volume of 50 µl of the recommended buffer with 0.3 µM trpseq2 and trpseq8 primers and 0.5 µl Takara e2TAK DNA polymerase for 30 cycles at 56° C. For restriction digestion, 5 µl of the PCR reaction was incubated with 2 units of SphI (New England Biolabs) in the recommended buffer in a total volume of 15 µl at 37° overnight and analyzed by gel electrophoresis. Sequencing of PCR products was performed by Beckman Coulter Genomics.

## Results

### Measuring incorporation of multiply-marked oligos

**Figure 2:**
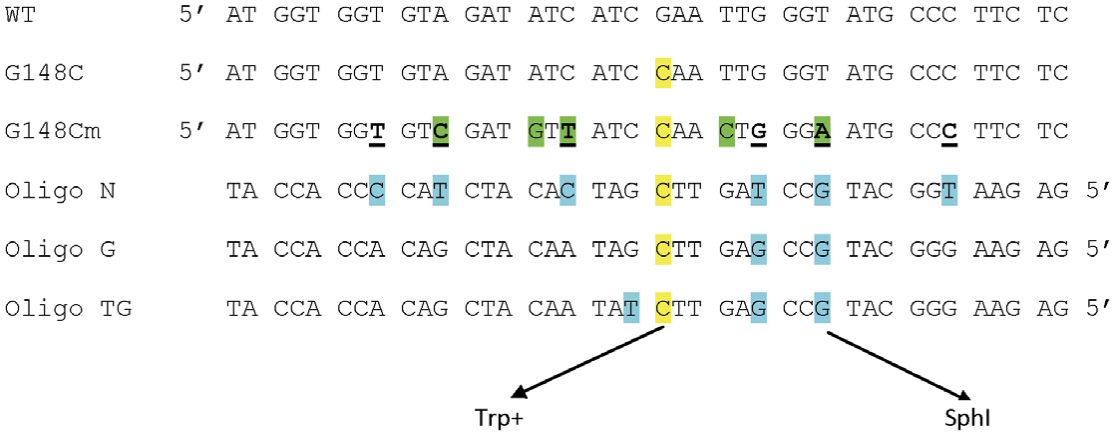
Sequences of *TRP5* mutant regions and oligonucleotides used for reversion analysis. The first line shows the wild-type sequence of *TRP5* from position 128 and the second line shows the G148C sequence, with the mutant C highlighted in yellow that must revert to G for *TRP5* function [20]. The G148Cm mutant contains several changes (highlighted in green) designed to create additional completely degenerate third codon positions, with those of interest underlined. Oligo N creates 7 mismatches (highlighted in yellow and blue) upon annealing with the G148Cm sequence, whereas Oligo G creates the indicated 3, and Oligo TG, 4 mismatches. The G in the oligos 12 nt from the 5′ end when incorporated creates a new SphI restriction site.

Even under optimal conditions in the absence of MMR, the frequency of oligo transformation is so low that one needs to be able to select for those cells that have successfully incorporated the oligo. We turned to the set of *trp5* point mutations we previously constructed [20]. These strains contain a mutation at either nucleotide position 148 or 149 of the *TRP5* gene and can only be reverted to the wild type phenotype by restoring the original *TRP5* sequence and thus have an extremely low rate of spontaneous reversion [20]. A potential problem was that the region surrounding the mutant base is highly conserved, constraining the location of any changed base. We therefore introduced several mutations into the region, maintaining with one exception the original amino acid sequence, but increasing the number of positions into which a different base could be substituted (Fig. 2). Because this mutant *trp5* G148Cm gene is placed close to a dependable origin of replication, and is present in both orientations relative to the origin (Fig. 1), we know which strand is replicated as leading and which as lagging and can reverse the replication strands by using a strain of opposite *TRP5* orientation [20].

For transformation, we used an oligo (N) that would create 7 mismatches when annealed to the G148Cm region (Fig. 2). In addition, in many cases oligos for transformation have been synthesized with phosphorothioate linkages at the ends for the purpose of increasing oligo stability in the cell [12], and so we also tested the effect of such linkages on marker incorporation, using oligos with 4 phosphorothioate linkages on the 5′ (5′ 4P) or 3′ (3′ 4P) ends to determine if phosphorothioate linkages would increase incorporation of nucleotides on the ends of the oligos. Because the oligos created only base-base mismatches in the genome, only MutSα should recognize the incorporated oligos. Therefore experiments were accomplished by transforming strains proficient (wt) or deficient (*msh6*) in MutSα, with individual revertants then being sequenced.

### Mismatch repair has little effect on the pattern of oligo incorporation

**Figure 3:**
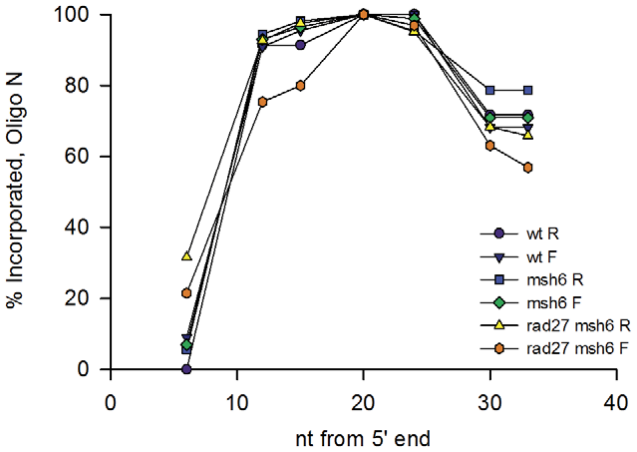
Nucleotides flanking the base inducing Trp+ reversion are usually incorporated in Trp+ revertants. Oligo N of Fig. 2 was transformed into G148Cm strains of the indicated genotype and Trp+ revertants sequenced to determine which oligo-induced mutations were present in each revertant, with the distance indicated from the 5′ end of the oligo. The indicated percentages are determined from approximately 48 revertants of each genotype. Trp+ revertants containing only the mutation at position 21 were assumed to be spontaneous revertants and were observed, rarely, and were not counted. In all experiments, oligos anneal to the leading strand of replication in the F orientation, and to the lagging strand in the R orientation.

Transformation of Oligo N was much more efficient into *msh6* strains deficient in MMR compared to MMR-proficient strains, but our interest was in determining what portions of the oligo were incorporated, rather than the absolute efficiency. The results of transforming Oligo N into *msh6* and wild-type G148Cm strains in both F and R orientations are shown in Fig. 3. All revertants included the C at position 21 of the oligos, as that is essential for a Trp+ phenotype. It was also evident that under any condition, bases within about 10 nt of that central position were incorporated at a high frequency, but generally less than 100%, and that the ends of the oligos were much less frequently incorporated. Sequencing also revealed that, as expected, there was no “skipping” of incorporated nucleotides (data not shown). The number of Trp+ revertants was higher with oligos annealing to the lagging strand (R) (data not shown), but as can be seen in Fig. 3 the pattern of incorporation was in general the same in both orientations. In addition, the pattern of incorporation wassimilar in the presence or absence of MMR (Fig. 3).

### The effect of phosphorothioate linkages on transformation

**Figure 4:**
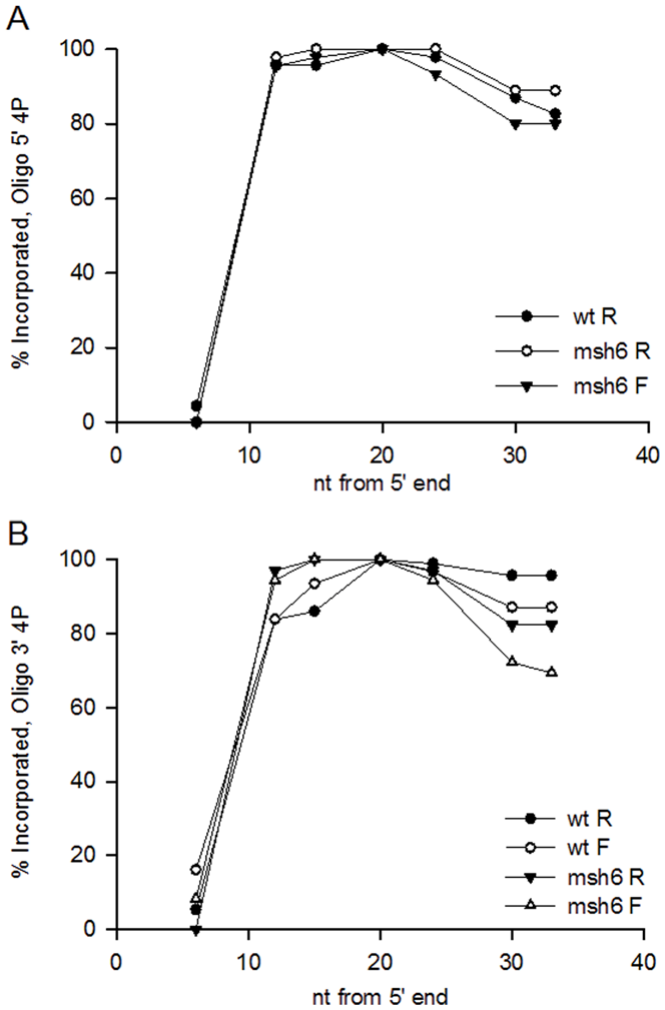
Phosphorothioate linkages make little difference in retention of oligo end sequences. Oligo N was transformed and results analyzed as in Fig. 3. (A) phosphorothioate linkages between the 4 nucleotides at the 5′ end of Oligo N. (B) phosphorothioate linkages between the 4 nucleotides at the 3′ end of Oligo N.

The loss of sequences from both ends of the oligo raised the question of whether the loss was due to exonucleolytic degradation that could be prevented by the use of phosphorothioate linkages that have been used in other oligo transformation experiments [12]. We therefore tested the effect of phosphorothioate linkages on marker incorporation, using oligos with 4 phosphorothioate linkages on either end. The results are shown in Fig. 4A and B. Somewhat surprisingly, the presence of phosphorothioate linkages at the 5′ end of the oligo made no noticeable difference in retention of sequences at the 5′ end of the oligo. However, the retention of nucleotides at the 3′ end of the oligo was modestly increased (Fig. 4A; compare to Fig. 3). The presence of phosphorothioate linkages at the 3′ end of the oligo also made no difference in retention of sequences at the 5′ end. However, at the 3′ end in wild-type strains, there was a noticeable increase in retention of nucleotides, but not in *msh6* strains (Fig. 4B). For example, retention of the marker 33 nt from the 5′ end was 87% for the modified and 68% for the unmodified in F wild-type strains, and 96% compared to 72% in R strains.

### Incorporation of 5′ oligo nucleotides is influenced by *RAD27*

If oligos serve as primers for replication, then an upstream Okazaki fragment on the lagging strand, or the replicating end on the leading strand, would have to eventually join up with the oligo-primed fragment and be ligated together. This reaction usually involves formation of a flap on the 5′ end of the primed fragment [25], and one explanation for the loss of nucleotides on the 5′ end of the oligos, and the lack of protection of the 5′ end by phosphorothioate linkages, was that the ends were being lost due to flap cleavage. The flap is usually cleaved by the Fen1 endonuclease (encoded by the *RAD27* gene in yeast) [25]; transformation by oligo N was therefore examined in *rad27 msh6* strains. As observed in Fig. 3, elimination of Rad27 led to increased retention of the 5′ ends of oligos on both the leading and lagging strands of replication (22% vs. 7% and 32% vs. 6% at nt 6 from the 5′ end for F and R strains respectively), indicating a role for Rad27 in loss of the ends.

### Mismatch repair processing of mismatches

**Figure 5:**
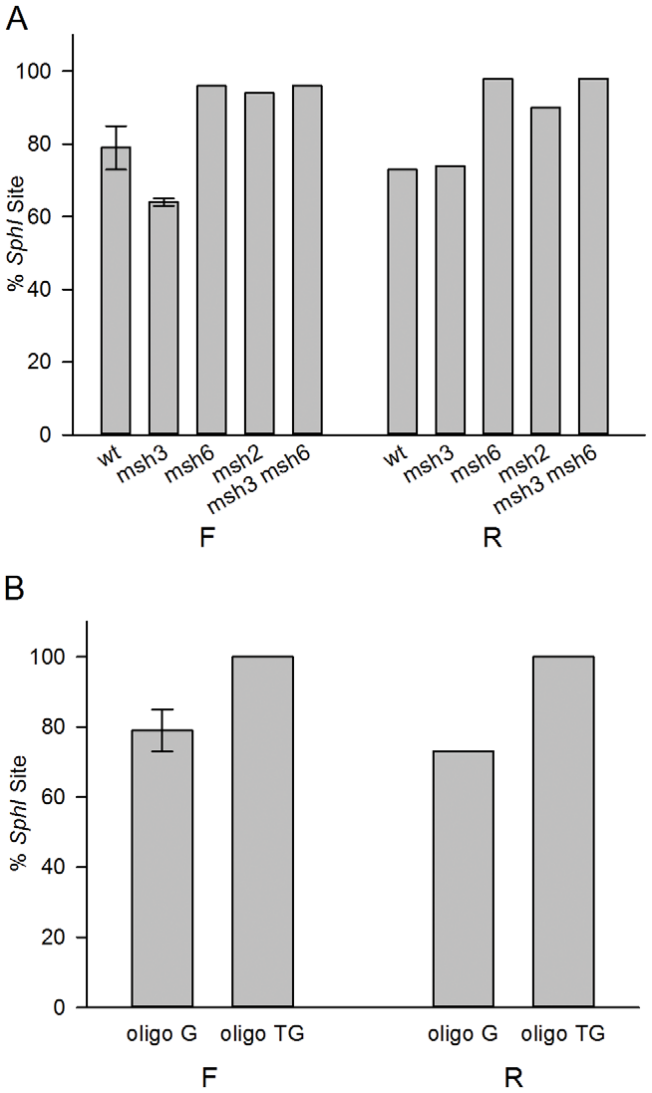
A C-C mispair is not always recognized by MMR . (A) Strains of the indicated genotypes in either the F or R orientation were transformed with Oligo G, and Trp+ revertants were selected and scored for the percentage that contained an SphI site created by a nucleotide in the oligo 8 nt 5′ of the C creating the Trp+ phenotype (Fig. 2). At least 40 revertants were scored in each phenotype. The error bars indicates the standard deviation observed from 2 independent experiments. (B) Wild-type strains were transformed with Oligo TG and analyzed as in (A). Data for Oligo G are from (A). Oligos anneal to the leading strand of replication in the F orientation, and to the lagging strand in the R orientation.

**Figure 6:**
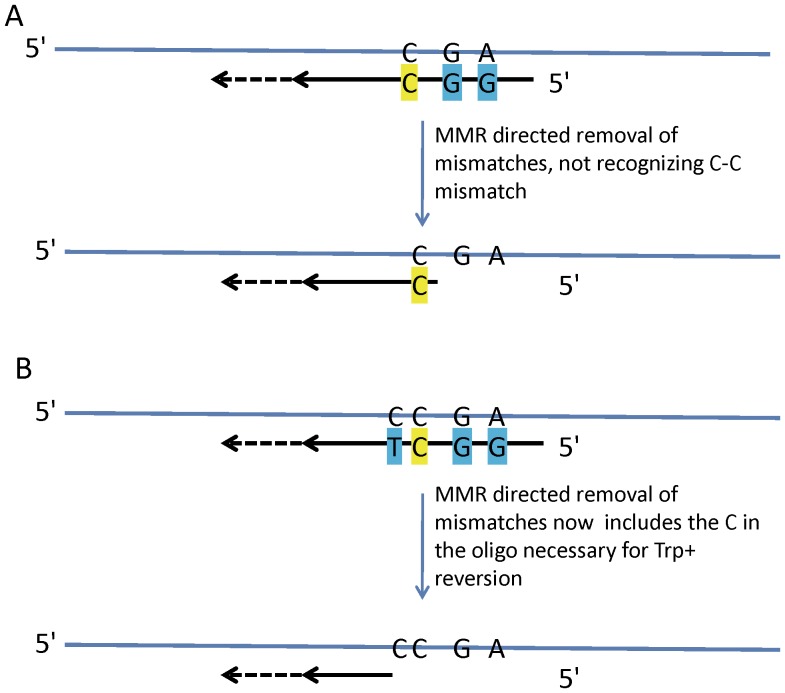
Models for action of MMR on oligos G and TG . (A) Oligo G anneals and primes replication as indicated. MMR should be the only system that would recognize the mismatches, and any nucleotides that remain after completion of the first round of replication should be replicated in the second round. If the C-C mismatch were poorly recognized by MMR, it is possible that it could remain after MMR action, as illustrated. (B) Oligo TG anneals and primers replication as indicated. In this case, even if the C-C mismatch were poorly recognized, MMR action should ensure removal of all mismatches.

The fact that the pattern of incorporation of oligo N was the same in the presence or absence of MMR suggested that those few oligos that escaped the action of MMR escaped completely. We designed a different oligo, oligo G, with only a few mismatches located centrally, illustrated in Fig. 2. That oligo creates a C-C mismatch to induce Trp reversion, a G-A mismatch 8 nt 5′ that would create a new SphI restriction site with retention of that part of the oligo, and a G-G mismatch between those two positions. The equivalent portion of oligo N is retained 91-94% of the time in both MMR-proficient and deficient strains, and we expected the same for oligo G. For oligo G, instead of sequencing, we could measure retention by SphI restriction site analysis of Trp+ revertants. As shown in Fig. 5A, strains deficient in MutSα all incorporated close to 100% of the central portion of oligo G. However, in wild-type strains or strains lacking only MutSβ (*msh3* strains), incorporation of that region varied from 64–79%. As illustrated in Fig. 6A, a revertant that does not have the SphI site but is Trp+ must have lost sequence from the 5′ end of the oligo during the initial round of replication. A likely explanation is that MMR recognizes the G/A and G/G mismatches, but recognizes the C/C mismatch more poorly [9], and in 25% of the cases, MMR-directed excision does not proceed through the C/C mismatch. In order to test this hypothesis, wild-type strains were transformed with Oligo TG (Fig. 2) that would create an additional mismatch 3′ of the C-C mismatch. With the additional mismatch created by Oligo TG, all Trp+ revertants contained the SphI site (Fig. 5B). Thus either the entire central portion of the oligo escapes MMR, or as illustrated in Fig. 6B, all of the mismatches are removed.

## Discussion

The use of oligos creating multiple mismatches with the genome has revealed valuable information, not only for their subsequent use, but also about replication and mismatch repair. If oligos serve as primers for replication, one might have expected them to transform only when annealed to the lagging strand of replication, which is replicated discontinuously, and not to the leading strand which is presumably replicated in a continuous fashion. However as we have found in yeast [5] and others in *E. coli* [14], although transformation is more efficient with oligos targeted to the lagging strand, it occurs with oligos targeted to the leading strand a few fold less in yeast and 30-fold less in *E. coli*. Although the efficiency of transformation is greatly reduced by MMR [5], the pattern of oligo incorporation is generally very similar in the presence or absence of MMR and whether the oligo was targeted to the leading or lagging strand of replication (Figs. 3 and 4). In all conditions examined, there was a clear asymmetry of retention of ends of the oligo, with the 5′ end being much less likely to survive than the 3′ end, as can be seen in Figs. 3 and 4. These results suggest that the mechanism of incorporation is independent of MMR, is similar on the leading and lagging strands of replication, and that usually all mismatches are recognized and eliminated by MMR, or none are.

One issue that arises from these results is the degree to which the leading strand is replicated in a continuous fashion. The DNA polymerase that replicates the leading strand, Pol ε, has at least *in vitro* a processivity that is not any greater than Pol δ, the polymerase that replicates the lagging strand [26]. In addition, there is considerable evidence both in *E. coli* [27] and in yeast [28] that replication on the leading strand can also be discontinuous. Thus it is perhaps not surprising that oligo transformation can occur on the leading strand. A large amount of evidence supports the view that lagging strand synthesis is done by Pol δ and that leading strand replication is initially carried out by Pol ε [29]. A recent model proposes that synthesis after any interruption on the leading strand is completed by Pol δ [28]. In that context it would be extremely interesting to know which polymerase was responsible for elongation of oligos targeted to the leading strand.

**Figure 7:**
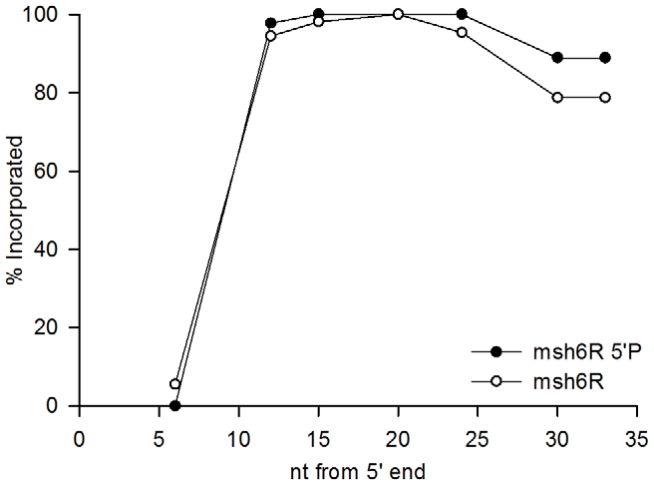
Comparison of retention of oligo N sequences with (5′P) and without phosphorothioate linkages at the 5′ end. Data are from Fig. 3 and 4A. Approximately the same difference is observed in *msh6F* strains.

The central core of the oligo was usually incorporated, but the 5′ end was rarely incorporated and about 1/3 of the time, 10 or more nucleotides on the 3′ end were not incorporated. The loss of nucleotides from the two ends appears to occur by fundamentally different mechanisms. Phosphorothioate linkages on the 5′ end of the oligo make no difference in the pattern of loss of the 5′ end, suggesting that the nucleotides are not removed exonucleolytically, or that the enzymes involved are not affected by the altered linkages. There is considerable evidence that phosphorothioate linkages do protect against a number of exonucleases [30], and so the lack of effect suggests that the 5′ end loss is not exonucleolytic. If the oligos serve as primers for replication, then ultimately the DNA primed by the oligo would have to be joined to DNA synthesized upstream as in normal Okazaki fragment maturation [25], [31]. Thus the loss of the 5′ end sequences could be due to the formation of a flap at the 5′ end with subsequent excision of the flap by Rad27 [25], [31], [32]; the increase in 5′ end sequences in a *rad27* strain indicates a role for Rad27 in flap excision of oligo sequences (Fig. 3). There are alternate pathways for fragment maturation not involving Rad27 [25]; the combination of those pathways is likely responsible for the major loss of oligo 5′ sequences, on both the leading and lagging strands. Although the leading strand of replication is generally replicated in a continuous manner, these results also indicate that new priming events on the leading strand are processed similarly to those on the lagging strand. It may be indicative of some difference between the two strands that on the leading strand the absence of Rad27 appears to effect the incorporation of some internal nucleotides in a manner different from that observed on the lagging strand (Fig. 3, compare incorporation of nucleotides 12 and 15 from the 5′ end in *rad27 msh6* F and R strains). A surprise is that although phosphorothioate linkages on the 5′ end do not make any appreciable difference in retention of the 5′ end, they do appear to have some affect on retention of the 3′ end (for the nucleotide at position 33, 89% retention versus 79% in the R orientation, and 80% vs. 71% in the F orientation) (Fig. 7). If the 5′ end were lost solely due to an endonucleolytic flap cleavage 5–10 bases from the 5′ end, one would not expect phosphorothioate linkages in the 5′-most 4 nucleotides to have any effect on maintenance of 3′ end sequences.

Phosphorothioate linkages do offer some protection on the 3′ end in wild-type, but little if any in *msh6* strains (Fig. 4B compared to Fig. 3). What could account for the loss of 3′ end nucleotides in MMR-proficient strains that is not observed when there are phosphorothioate linkages at the 3′ end? It has been demonstrated that phosphorothioate linkages protect against at least some DNA polymerase proofreading activities [33]. This suggests that some 3′ nucleotides may be lost by MMR-directed excision from the 3′ end, possibly by the 3′ proofreading exonuclease of the replicating polymerase [32]. This could be an indication of a limited type of MMR function, involved in only surveillance of the 3′ end, as there is no overall difference in pattern of unmodified oligos with or without MMR (Fig. 3). The 3′ end of transforming oligos is not always lost, as we have been able to induce transformants using oligos in which the 3′ nucleotide has to be incorporated for reversion; this process can be quite efficient when the terminal mismatch is well tolerated, such as an 8-oxoG-A mismatch (results not shown).

There have been different conclusions on the use of phosphorothioate bonds in oligos used for transformation. The original experiments on transformation in yeast used unmodified oligos [1]–[3]. Phosphorothioate linkages were later found to increase transformation in yeast by several fold in a different lab [12]. In mammalian cells, the situation is complex [7]. The use of oligos protected with phosphorothioate linkages at both ends induced cell cycle arrest and double-strand breaks [34], [35]. In one study it was found that oligos with phosphorothioate linkages at both ends gave greater transient correction than oligos with unmodified ends, but gave significantly fewer stable colonies [36]. In the absence of *MSH2*, transient correction was highest for unmodified oligos, followed in decreasing order by oligos modified at the 3′, 5′, or both 3′ and 5′ ends, but for those experiments relative viable formation of colonies was not reported [36]. More recently it was found that transformation of *msh2* cells was more efficient with unmodified oligos than oligos with phosphorothioate linkages at both ends and that the unmodified oligos created much less cell cycle disturbance [15]. That would suggest that, at least in mammalian cells, oligos with phosphorothioate linkages at the ends can lead to double-strand breaks and cell cycle disruption and therefore fewer viable transformed colonies than the use of unmodified oligos, although the reason for the difference was not understood [7]. A recent report studying oligo transformation in HeLa cells found that toxicity was correlated with increasing number of phosphorothioate bonds, possibly due to stimulation of cellular immunity, and that a few internal phosphorothioate linkages 3′ to the mismatch were most effective in creating stable transformants [37].

What might be the cause of cell-cycle arrest and double strand breaks observed in mammalian cells due to oligo transformation with oligos containing phosphorothioate linkages? Based on our observations, it appears that phosphorothioate linkages on the 5′ end could be problematical in replication fragment joining. It is clear that whatever process is used to join the 5′ end of the oligo into the completed replicated strand involves some sort of 5′ end processing, and the fact that a change in such processing could cause even slight differences on incorporation of 3′ end sequences, as observed in Fig. 7, suggests a significant change in oligo incorporation. Our experiments do not measure the incorporation of bases at the very 3′ end of the oligo, but it is clear that there is a tendency to lose bases at the 3′ end, and it may be that the usual method of primer extensive could involve a small degree of 3′ resection, which would be prevented by phosphorothioate linkages, again partially disrupting the normal incorporation.

The interesting exception to a similar pattern of transformation in wild-type and *msh6* strains was provided by oligo G, where in wild-type, but not *msh6* strains, nucleotides close to the center of the oligo were lost in 25% of transformants (Fig. 5A). This loss was shown to be likely due to the occasional failure of MMR to recognize a C-C mismatch, as a well-recognized mismatch created just to the 5′ side of the C-C mismatch (oligo TG) resulted in retention of all oligo nucleotides in Trp+ revertants (Fig. 5B). The location of the excised nucleotides relative to the retained nucleotides showed that in this case MMR-directed excision was from the 5′ end of the oligo and that excision must not have proceeded more than 4 nucleotides past the recognized mismatches or else as can be seen in Fig. 6A, the C-C mismatch would have been removed resulting in no Trp+ revertants. Recent work analyzing single-base mispairs created by polymerase errors found that errors created by Pol α were corrected more efficiently by MMR than errors created by Pol δ, and it was hypothesized that the difference might be due to the use of the 5′ end of the Okazaki fragment as a strand discrimination signal [38]. Our results are consistent with MMR-directed excision from the 5′ end of a replicating segment, and further indicate that such excision likely stops directly after the recognized mispair.

In order to make optimum use of oligo transformation, it is important to understand the parameters of oligo incorporation into the genome. As part of this work, we have shown that oligos can be used to introduce multiple changes into the genome simultaneously. With oligos that are 40 nt in length, there is a central core of greater than 15 nt that is almost always incorporated. Longer oligos would be expected to have a correspondingly longer core of nucleotide incorporation. One factor that is clearly important for the incorporation of a given part of an oligo is its distance from either end of the oligo. MMR represents a strong barrier to oligo transformation, and approaches involving transient inactivation of MMR appear most promising in circumventing oligo rejection by MMR [7], [39]. Even in the absence of MMR, however, in yeast only a small fraction of cells are transformed by a given oligo [5], [6], for reasons that are not entirely clear. The mechanism of incorporation suggests that for a given cell, there would only be a short window for transformation in which the region of interest was being replicated and had a single-stranded region accessible for oligo annealing. Although the stability of the oligo to cellular exonucleases could be an issue in efficiency, protection of the ends with phosphorothioate linkages seems to introduce other problems, and has not led to noticeable increases in transformation efficiency in our hands. Another possibility would be that oligos were effectively being inactivated by protein binding or transport from the nucleus. Understanding the remaining causes of low transformation efficiency will be important for any potential therapeutic uses.

## Supporting Information

Table S1 **Strain genotypes.**
(DOCX)

Table S2 **Oligo sequences.**
(DOCX)
