# Supplementary material for: Transformation with Oligonucleotides Creating Clustered Changes in the Yeast Genome
Source: PLoS One. 2012 Aug 14;7(8):e42905. doi: 10.1371/journal.pone.0042905 (PMC3422593; doi:10.1371/journal.pone.0042905)
Supplement: Table S1 — Strain genotypes. (DOCX) [file pone.0042905.s001.docx]

Table S1. Strain genotypes.

| **Strain number** | **Trp5 orientation** | **Relevant Genotype** |
| --- | --- | --- |
| GCY2196 | F | *MATα his3Δ200 ura3-52 leu2 Δ1 trp5G148Cm lys2CT_1265_GA* |
| GCY2297 | R | *MATα his3 Δ200 ura3-52 leu2 Δ1 trp5G148Cm lys2CT_1265_GA* |
| GCY2335 | F | GCY2196 *msh6 Δ::HygMX* |
| GCY2336 | R | GCY2297 *msh6 Δ::HygMX* |
| GCY2414 | F | GCY2196 *msh2 Δ::hphMX4* |
| GCY2408 | R | GCY2297 *msh2 Δ::hygMX* |
| GCY2458 | F | GCY2196 *msh3 Δ::KanMX* |
| GCY2459 | R | GCY2297 *msh3 Δ::KanMX* |
| GCY2588 | F | GCY2196 *msh3 Δ::KanMX* *msh6 Δ::HygMX* |
| GCY2589 | R | GCY2297 *msh3 Δ::KanMX* *msh6 Δ::HygMX* |
| GCY2615 | F | GCY2196 *msh6 Δ::HygMX*  *rad27 Δ::KanMX* |
| GCY2627 | R | GCY2297 *msh6 Δ::HygMX*  *rad27 Δ::KanMX* |
