# Supplementary material for: Transformation with Oligonucleotides Creating Clustered Changes in the Yeast Genome
Source: PLoS One. 2012 Aug 14;7(8):e42905. doi: 10.1371/journal.pone.0042905 (PMC3422593; doi:10.1371/journal.pone.0042905)
Supplement: Table S2 — Oligo sequences. (DOCX) [file pone.0042905.s002.docx]

Table S2. Oligo sequences.

| **Primer** | **Sequence** |
| --- | --- |
| oligo N | GAGAATGGCATGCCTAGTTCGATCACATCTACCCCACCAT |
| oligo G | GAGAAGGGCATGCCGAGTTCGATAACATCGACACCACCAT |
| oligo TG | GAGAAGGGCATGCCGAGTTCTATAACATCGACACCACCAT |
| LYS2TCARev | CCAACCCTATCTTTCACATCAGGTTCCGAAGGTATTCCTA |
| trpseq2 | CAGGAACGCCTTGGTCACAT |
| trpseq8 | ATGGGTACGGTAACACCTTC |
